# Improving the Oxygen Evolution Reaction on Fe_3_O_4_(001) with Single-Atom Catalysts

**DOI:** 10.1021/acscatal.3c00337

**Published:** 2023-03-24

**Authors:** Enrico Bianchetti, Daniele Perilli, Cristiana Di Valentin

**Affiliations:** † Dipartimento di Scienza dei Materiali, Università di Milano Bicocca, Via Roberto Cozzi 55, 20125 Milano, Italy; ‡ BioNanoMedicine Center NANOMIB, Università di Milano Bicocca, Via Raoul Follereau 3, 20900 Monza, Italy

**Keywords:** density functional theory, hybrid functional, computational electrochemistry, oxygen evolution reaction, water splitting, magnetite, single-atom catalysts, transition-metal adatoms

## Abstract

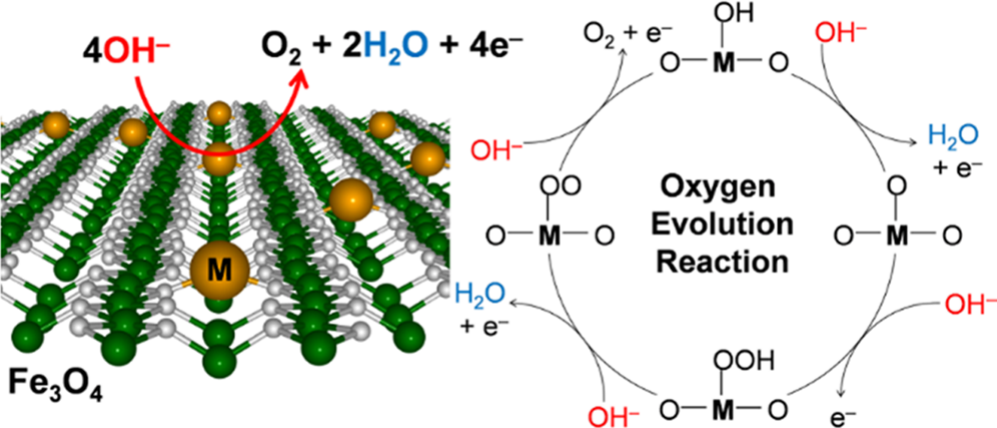

Doping magnetite surfaces with transition-metal atoms is a promising strategy to improve the catalytic performance toward the oxygen evolution reaction (OER), which governs the overall efficiency of water electrolysis and hydrogen production. In this work, we investigated the Fe_3_O_4_(001) surface as a support material for single-atom catalysts of the OER. First, we prepared and optimized models of inexpensive and abundant transition-metal atoms, such as Ti, Co, Ni, and Cu, trapped in various configurations on the Fe_3_O_4_(001) surface. Then, we studied their structural, electronic, and magnetic properties through HSE06 hybrid functional calculations. As a further step, we investigated the performance of these model electrocatalysts toward the OER, considering different possible mechanisms, in comparison with the pristine magnetite surface, on the basis of the computational hydrogen electrode model developed by Nørskov and co-workers. Cobalt-doped systems were found to be the most promising electrocatalytic systems among those considered in this work. Overpotential values (∼0.35 V) were in the range of those experimentally reported for mixed Co/Fe oxide (0.2–0.5 V).

## 1 Introduction

Renewable resources to produce electricity, such as solar or wind power, are emerging alternatives to decrease the use of fossil fuels. However, their fluctuating nature imposes great challenges since energy storage solutions are required to compensate for the downtimes in production. Water electrolysis, also known as electrochemical water splitting, is a promising technology for the storage of the surplus electric energy via conversion into chemical energy in the form of hydrogen gas fuel.

The process comprises two half-cell reactions separated by a membrane: the cathodic hydrogen evolution reaction (HER)
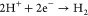


and the anodic oxygen evolution reaction (OER)
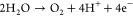


under acidic conditions. The OER is more kinetically sluggish because it is a four-electron transfer reaction compared with the HER that requires only two electrons. Therefore, the key process governing the overall efficiency of water electrolysis is the OER, which is often referred to as the bottleneck reaction for the hydrogen production from water.

State-of-the-art OER electrocatalysts contain noble metals, e.g., Ru, Ir, and Pt, and work in acidic media, showing noticeable stability and activity.^1^ However, such catalysts are not convenient for large-scale applications because of the scarcity and cost of the precious metals involved in their realization. On the contrary, in alkaline solution, many abundant and inexpensive metals, e.g., Fe, Co, and Ni, and their alloys show comparable catalytic performance to noble metals.^2^

Nonetheless, alkaline water electrolysis has long been considered inefficient compared to the acidic one because of the poor performance of the hydroxide-conducting polymer electrolyte membranes (PEMs) separating the two half-cells compared to the proton-conducting ones. However, recent studies have suggested that it is possible to reach similar or even higher activities with alkaline cells through the optimization of the hydroxide-conducting PEMs that are currently used in existing systems.^3,4^ Therefore, the search for good OER catalysts in alkaline media is an active field of research, where transition-metal oxides, hydroxides, and oxyhydroxides containing Ni, Co, and Fe have been proposed as suitable candidates.^5−17^

Recently, magnetite (Fe_3_O_4_) has attracted the interest of the scientific community as a simple model surface where to investigate the complex OER mechanism since this is still under debate when the newly emerging inexpensive transition-metal oxide catalysts are involved. In particular, the Fe_3_O_4_(001) surface, besides being the most stable and exposed facet in magnetite nanostructures,^18−22^ is also the best understood and well-defined in terms of the atomic structure, both in vacuum and water environment, which is a crucial aspect when trying to determine what are the reaction intermediates on the surface along the reaction path.

The stacking sequence in the [001] direction consists of A layers that contain Fe_Tet_ and B layers that contain O and Fe_Oct_. Over time, different atomic models have been proposed for the (001) surface. In 2005, based on density functional theory (DFT) calculations, Pentcheva et al.^23^ proposed a clean B layer termination, called distorted bulk truncation (DBT) model, thermodynamically more stable than other previously suggested configurations.^23,24^ In 2014, through a combined experimental and theoretical study, Bliem et al.^25^ proposed a new reconstructed surface model called subsurface cation vacancy (SCV) characterized by a B layer-terminated Fe_3_O_4_(001) surface with an extra interstitial Fe_Tet_ in the second layer, replacing two Fe_Oct_ that are removed from the third layer, per each (√2 × √2) R45° unit cell. The SCV model shows a much better agreement with the experimental findings^25−28^ and is found to be more stable than the DBT model in vacuum and under water exposure up to elevated temperature values, i.e., more than 700 K.^29−33^ Furthermore, the structure of the Fe_3_O_4_(001)/water interface has also been elucidated in recent years.^29,34,35^

Fe_3_O_4_ was experimentally reported to be characterized by a good stability and a fair activity toward OER. Through low-energy electron diffraction (LEED), scanning tunneling microscopy (STM), and atomic force microscopy (AFM) measurements, Müllner et al.^36^ did not register any change in the SCV surface morphology after having increased the pH up to the values that are typically used for the water oxidation by transition-metal oxides in alkaline conditions. They also observed that the SCV surface morphology was unchanged after having performed several cyclic voltammetry scans at an overpotential value of 0.48 V. Similarly, also Grumelli et al.^37^ confirmed the stability of the SCV surface reporting an overpotential of 0.44 V. In less oxidizing potential conditions (but still in the OER regime), they also succeeded in stabilizing the unreconstructed DBT surface, for which they measured an overpotential value of 0.49 V.

From the computational point of view and by means of DFT + *U* calculations, Righi et al.^33^ have recently investigated the stability and the electrochemical performance toward OER for both SCV and DBT models of the Fe_3_O_4_(001) surface. First, they studied the interaction of these two surface models with water molecules and their relative stability in an aqueous and electrochemical environment in a wide range of oxygen chemical potentials. Second, they proposed and investigated two different mechanisms for the OER taking place at the Fe_3_O_4_(001) surface. One mechanism is based on the conventional adsorbate evolution mechanism (AEM), which implies the O–O bond formation through the attack of a water molecule or a hydroxide ion (depending on the pH) on an oxo group. The other mechanism is based on the lattice oxygen-mediated mechanism (LOM), which involves oxygen atoms originally belonging to the oxide surface, rather than to the adsorbates, in the O–O bond formation. The two mechanisms were found to be competitive on both SCV and DBT surfaces. In 2014, Li and Selloni^38^ performed a similar investigation on the DBT surface (SCV was proposed only few months later^25^), considering only the LOM mechanism, and obtained analogous results.

On the reconstructed Fe_3_O_4_(001) surface, transition-metal adatoms have been successfully isolated, exploiting the presence of arrays of strongly binding sites (periodicity 0.84 nm along the [110] direction), on which metal atoms coordinate two surface lattice oxygen atoms.^21,25,27,39^ Transition-metal atoms are found to be stable against thermal sintering on Fe_3_O_4_, but some of them, such as Ti,^40^ Mn,^40^ Co,^40,41^ Ni,^40,42^ Zr,^40^ Rh,^43^ Pd,^44^ and Ir,^45^ between room temperature and 500 K, tend to diffuse, and, in some cases, agglomerates in clusters or, in others, fill the Fe_Oct_ vacancies and become incorporated into the subsurface layers, leading to the restoring of a DBT-like surface structure. On the contrary, metals such as Cu,^46^ Ag,^46,47^ Au,^25,27^ and Pt^48,49^ do not become incorporated in the spinel lattice and remain stable as adatoms at least until the reconstruction is thermally lifted at 700 K.

Mixed Fe oxides being among the most promising materials for OER electrocatalysts,^6,9,11,12,14−17^ with rather low overpotentials, one would reasonably expect that incorporating or loading as adatoms transition-metal atoms at the Fe_3_O_4_(001) surface, forming so-called single-atom catalyst (SAC) species, could improve the overall catalytic performance of magnetite. This is still an open question, and whether having few transition-metal adatoms on the surface would be as efficient as a mixed metal oxide is still to be proved. A recent experimental study on Ni adatoms loaded on the Fe_3_O_4_(001) surface does not seem to corroborate this hypothesis for the case of Ni.^50^

To the best of our knowledge, no theoretical mechanistic investigation has been yet presented elucidating the performance, together with the reaction pathways, of transition-metal single atoms at the Fe_3_O_4_(001) surface toward OER. Up to now, existing computational studies are limited to the investigation of the pristine Fe_3_O_4_(001) surface, as detailed above.

In this work, we present a thorough study, based on the wide set of hybrid density functional theory calculations (see Section 2 for the details on methods and models), where we investigate the potential of Fe_3_O_4_(001)-supported SACs as electrocatalytic systems for OER and use the pristine DBT Fe_3_O_4_(001) surface as the reference system. Different mechanisms are considered as described in Section 3. In Section 4, first we assess the chosen computational setup by comparing our results on the pristine DBT Fe_3_O_4_(001) surface with those already present in the literature.^33,38^ Second, we discuss structural, electronic, and magnetic properties of isolated transition-metal atoms (Ti, Co, Ni, and Cu) on the Fe_3_O_4_(001) surface. In particular, Ti atoms are considered only when incorporated in the DBT-like surface because experiments indicate that they are not stable as adatoms, even at room temperature.^40^ Co and Ni dopants are studied both as adatoms on an SCV surface model and as incorporated in the DBT-like surface model because Co^40,41^ and Ni^40,42^ atoms become partially incorporated at room temperature and at slightly higher temperature (i.e., ca 400 K), respectively. Cu dopants are studied only as adatoms on the SCV surface model because Cu is not observed to become incorporated at any temperature where SCV is stable.^46^ Finally, for all of the designed transition-metal-doped surface models, we analyze OER intermediates and reaction pathways to evaluate their catalytic performance in terms of computed Gibbs free energy profiles and theoretical overpotentials. On the basis of these results, we will be able to establish whether small quantities of single-atom catalysts at the Fe_3_O_4_(001) surface perform as good as mixed Fe oxides.

## 2 Methods and Models

Hybrid DFT calculations (HSE06)^51,52^ were carried out using the CRYSTAL17 package^53,54^ to study the structural, electronic, magnetic, and thermodynamic properties of all systems under investigation. For the validation against experimental data of the standard hybrid functional HSE06 as a robust theoretical approach to describe structural, electronic, and magnetic properties of magnetite system, please refer to ref (55) and the corresponding Supporting Information, where the effect of reducing the fraction of the exact exchange was analyzed, in comparison with B3LYP calculations and PBE+U calculations with different U values. The Kohn–Sham orbitals were expanded in Gaussian-type orbitals: the all-electron basis sets are H-511G(p1), O-8411G(d1), (Ti, Fe, Co, Ni)-86411G(d41), and Cu-864111G(d41). The convergence criteria of 10^–7^ hartree and 4.5 × 10^–5^ hartree/bohr for total energy and forces, respectively, were used during the self-consistent field, geometry optimization, and vibrational frequency calculations. For these calculations, the irreducible Brillouin zone was sampled with a 3 × 3 × 1 *k*-point grid generated with the Monkhorst–Pack scheme.^56^ For the calculation of the projected density of states (PDOS), a denser *k*-point mesh of 6 × 6 × 1 was used. The PDOS were analyzed through the band center of mass (COM) descriptor that was computed using the formula
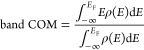
1where *E* is the energy, *E*_F_ is the Fermi energy (which is set to 0), and ρ(*E*) is the electronic density of states.^57−60^

Vibrational frequencies of each isolated molecule (H_2_, O_2_, and H_2_O), isolated surface, and adsorbate bound to the surface were calculated at the Γ point within the harmonic approximation. To do so, numerical Hessian matrices were constructed from finite displacements and force components on each atom. The adsorbed intermediates, as well as the transition-metal and oxygen atoms nearest to the intermediates themselves, were displaced by ±0.003 Å in all three Cartesian directions from their equilibrium positions. The resulting Hessian matrix then was diagonalized to yield vibrational frequencies corresponding to each mode. Enthalpic and entropic contributions were calculated at standard-state conditions using the ideal gas, rigid rotor, and harmonic approximation to evaluate, respectively, the translational, rotational, and vibrational terms for each isolated molecule (H_2_, O_2_, and H_2_O) along with only vibrational terms for the isolated surface and adsorbate bound to the surface.

The OER Gibbs free energy profiles were derived within the framework of the computational hydrogen electrode (CHE)^61^ using the standard hydrogen electrode (SHE) at pH = 14 as a reference.^62^ The proton and electron Gibbs free energy *G*(H^+^ + e^–^) can be rewritten as
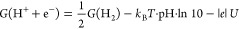
2where *G*(H_2_) is the Gibbs free energy of H_2_ and |*e*|*U* is the applied electrode potential per electron. Being at pH = 14, because experiments for the OER on Fe_3_O_4_ are conducted under very alkaline conditions,^36,37,50^ the release of H^+^ and e^–^ is replaced by the consumption of OH^–^ and the release of e^–^, whose Gibbs free energy can be written as
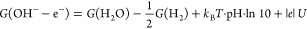
3where *G*(H_2_O) is the Gibbs free energy of H_2_O and *G*(OH^–^ – e^–^) is the Gibbs free energy of the hydroxide ion and electron pair (the minus sign indicates that one species is consumed and the other is released). *G*(H_2_O) was computed at 0.035 bar because, at this pressure, gas-phase water is in equilibrium with liquid water at 300 K, i.e., *G*(H_2_O_liquid_) = *G*(H_2_O_gas,p = 0.035bar_).^61^ The theoretical overpotential (η) is defined for a given mechanism in which the most endoergic elementary step involves a redox reaction. It is calculated by subtracting the cumulative free energies of all of the steps in the mechanism divided by the number of electrons involved (here, four), which gives the theoretical thermodynamic potential (*U*_0_) from the potential of the most endoergic redox step. The onset potential (*U*^onset^) is given by the sum of the thermodynamic potential and overpotential (*U*^onset^ = *U*_0_ + η), and it represents the minimum applied potential required to release the products.

Both DBT and SCV surface models^23,25^ were used to model pristine Fe_3_O_4_(001) surfaces and were constructed as a (1 × 1) 17-layer slab with inversion symmetry, in line with previous works by some of us.^29,35,63^ The atoms in the central five layers of the slab were kept fixed to their bulk position, whereas the atoms in the other layers were fully relaxed. For the deposition or incorporation of transition-metal atoms and for the OER intermediates, atoms were put on both sides of the slab.

**Figure 1:**
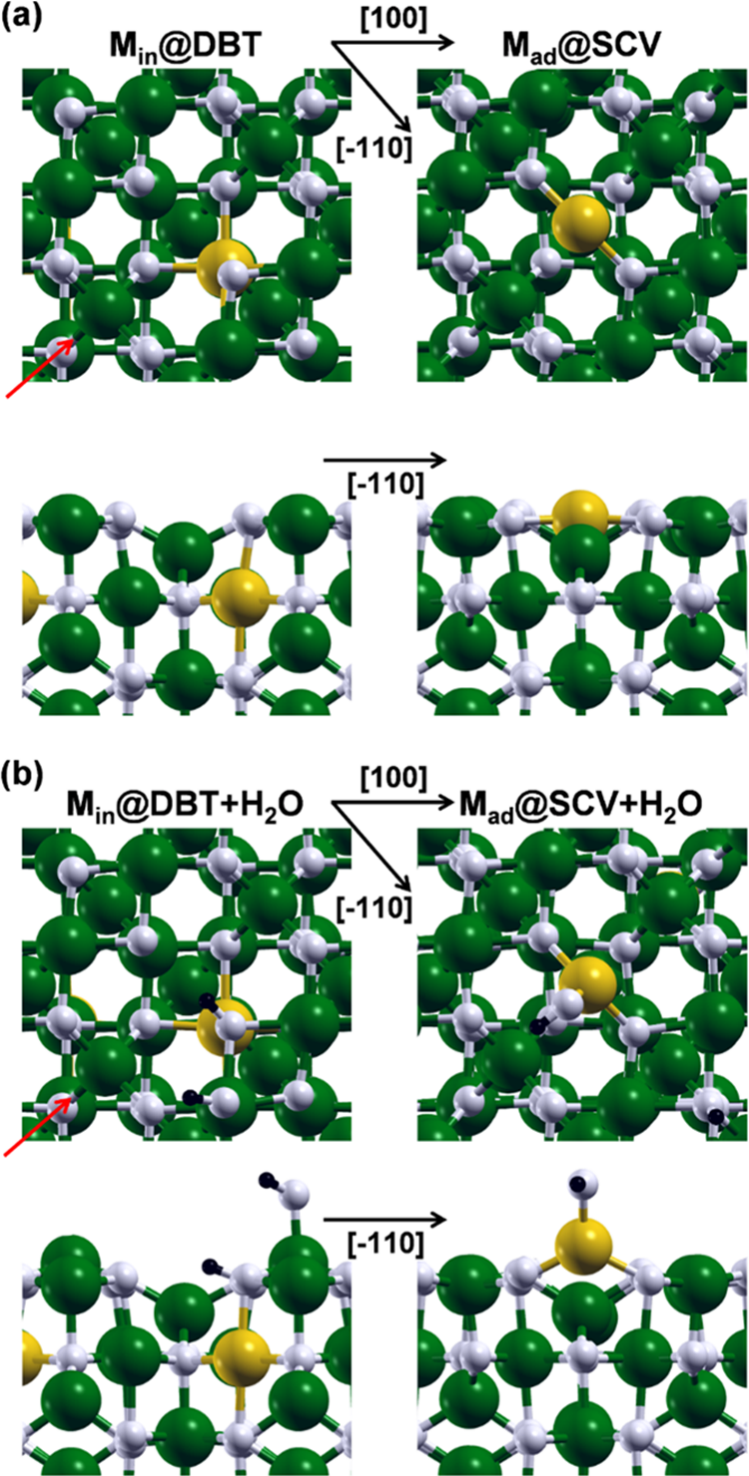
Top (first row) and side (second row) views of a transition-metal atom incorporated in the DBT surface (on the left) and loaded as adatom on the SCV surface (on the right) in the absence (a) and in the presence (b) of a dissociated water molecule. The green, white, black, and yellow beads represent Fe, O, H, and doping transition-metal atoms, respectively. For the left panels, M = Ti and Co. For the right panels, M = Co, Ni, and Cu. The black arrows indicate the crystallographic directions, whereas the red arrows indicate the direction of the side views.

Incorporated Ti and Co atoms were modeled by substituting a Fe_Oct_^III^ ion in the third layer of the DBT surface, as shown in the left panels of Figure 1a. These models are named Ti_in_@DBT and Co_in_@DBT, respectively. Co, Ni, and Cu adatoms on the SCV surface were modeled as bound to two surface lattice oxygen atoms (see right panels in Figure 1a), which have been previously recognized to be the most reactive.^25,27,64−66^ These models are named Co_ad_@SCV, Ni_ad_@SCV, and Cu_ad_@SCV, respectively.

As an approximate description of the presence of the solvent, one dissociated water molecule was adsorbed on the slabs:^38,67,68^ the OH fragment was adsorbed on an exposed fivefold coordinated Fe_Oct_^III^ (in the case of clean DBT, Ti_in_@DBT, and Co_in_@DBT, left panels in Figure 1b) or on an adatom (in the case of Co_ad_@SCV, Ni_ad_@SCV, and Cu_ad_@SCV, right panels in Figure 1b), whereas the H fragment was adsorbed on an exposed surface oxygen nearby. The adsorption energy (*E*_Ads_) for the dissociated water molecule was calculated as follows
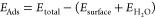
4where *E*_total_ is the total energy of the whole system (surface and adsorbed water), *E*_surface_ is the energy of the isolated Fe_3_O_4_(001) surface, and *E*_H_2_O_ is the energy of one isolated water molecule.

## 3 Mechanism of OER on a Metal Oxide Surface

As mentioned in Section 1, it is widely accepted that OER on a transition-metal oxide can proceed through two different reaction paths: the conventional AEM and the LOM.^69^ The AEM is typically assumed to involve four concerted proton-coupled electron transfer (PCET) reactions centered on the metal ion, and the O–O bond formation goes through the addition of a water molecule (in acidic environments) or a hydroxide ion (in alkaline environments) on an oxo group previously obtained from the deprotonation of an adsorbed water molecule or hydroxide ion.^70−72^ Differently, the LOM may involve nonconcerted proton–electron transfers, and the reaction steps do not proceed only on the metal site, as in the case of AEM, but they also involve oxygen atoms originally belonging to the oxide surface for the O–O bond formation.^73,74^

**Figure 2:**
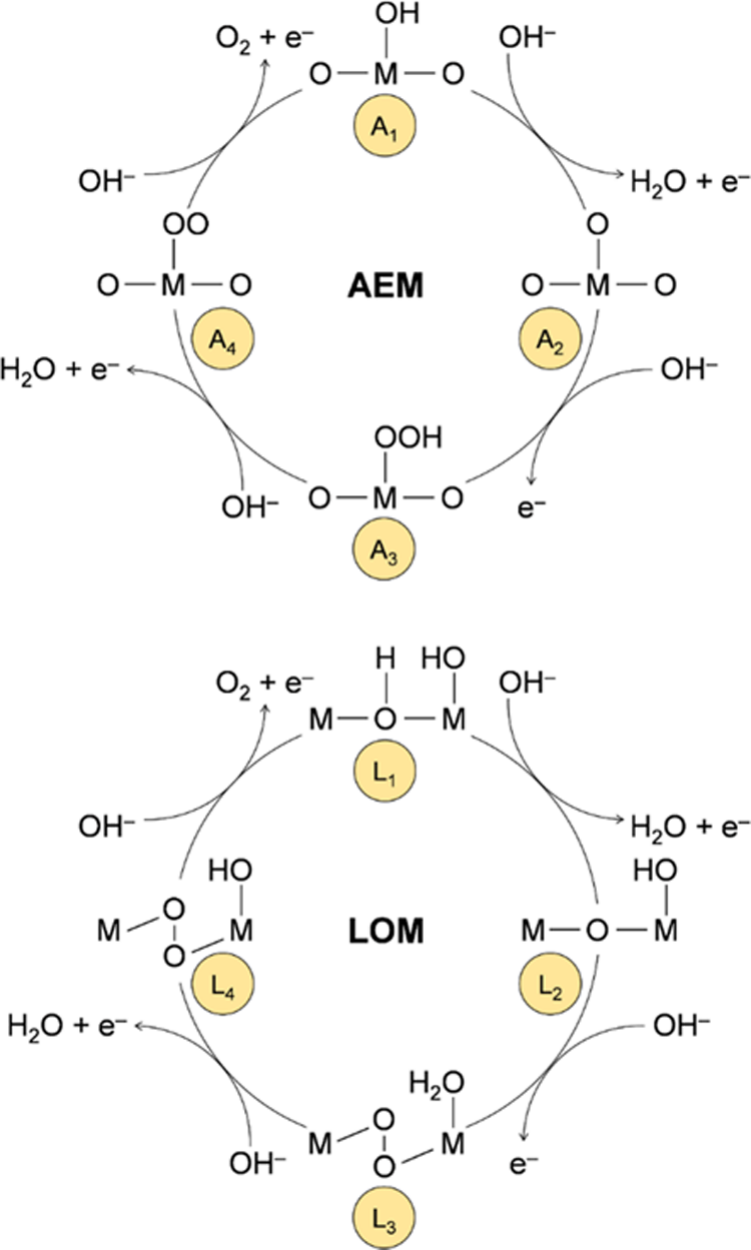
Schematic representation of the studied OER mechanisms, namely, AEM on top and LOM on bottom.

In this work, the AEM is studied according to the conventional scheme shown in Figure 2. The starting point is the OH adsorbed on an exposed metal site (A_1_ in Figure 2), and the first step consists in its dehydrogenation. As a second step, the adsorbed O (A_2_ in Figure 2) undergoes an addition by a hydroxide ion forming the hydroperoxo OOH (A_3_ in Figure 2). As a third step, the adsorbed OOH is dehydrogenated to form the OO superoxo (A_4_ in Figure 2) on the same metal site. As a fourth and final step, O_2_ is released, and the catalyst is re-established through the adsorption of a hydroxide ion on the metal site.

The LOM is studied according to the scheme proposed by Li & Selloni^38^ for the NiFe_2_O_4_(001) surface, as shown in Figure 2. The starting point is the OH group formed by the adsorption of the hydrogen dissociated from water to an oxygen of the surface (L_1_ in Figure 2). The first step consists in the dehydrogenation of such OH, leading to L_2_ in Figure 2. As a second step, the OO peroxo is formed within the surface lattice and, simultaneously, the OH adsorbed on an exposed fivefold coordinated Fe_Oct_^III^ is hydrogenated to form a water molecule (L_3_ in Figure 2). As a third step, this water molecule is dehydrogenated leading to L_4_ in Figure 2. As a fourth and final step, the catalyst is restored through the adsorption of a hydroxide ion into the lattice oxygen vacancy left by the O_2_ release. Different intermediates were investigated, but they resulted to be less favored than those just described above.

We wish to mention that, for both mechanisms, all of the steps were treated as PCET reactions, even if at alkaline anodic conditions—that characterize OER at transition-metal oxide surfaces—one could expect that some proton transfers are spontaneously decoupled from electron transfers. For instance, it is reasonable to suppose that the first LOM step (L_1_ → L_2_) occurs as a sequential deprotonation–oxidation process since a proton on a surface oxygen is probably a very labile species. See ref (74) for further discussion about nonconcerted proton–electron transfer steps in the LOM.

The AEM is studied for all of the models defined in the previous section. On the contrary, the LOM is investigated only for the DBT-based models. The reason for this choice will be discussed in detail below.

The nomenclature is defined according to the following rules: the name starts with the type of mechanism (AEM or LOM) and then proceeds with the surface model used (DBT or SCV or M_in_@DBT or M_ad_@SCV), followed by an additional formula of −MO_nc_H, where M indicates the metal atom where the OH species taking part in the OER is specifically adsorbed and the subscript nc defines the coordination number of the oxygen involved, i.e., 1c for an O atom in the hydroxide ion and 3c for a lattice oxygen. In the case of O_1c_, the reaction proceeds via AEM, while in the case of O_3c_, it proceeds via LOM.

## 4 Results and Discussion

### 4.1 OER on a Clean DBT Surface

**Figure 3:**
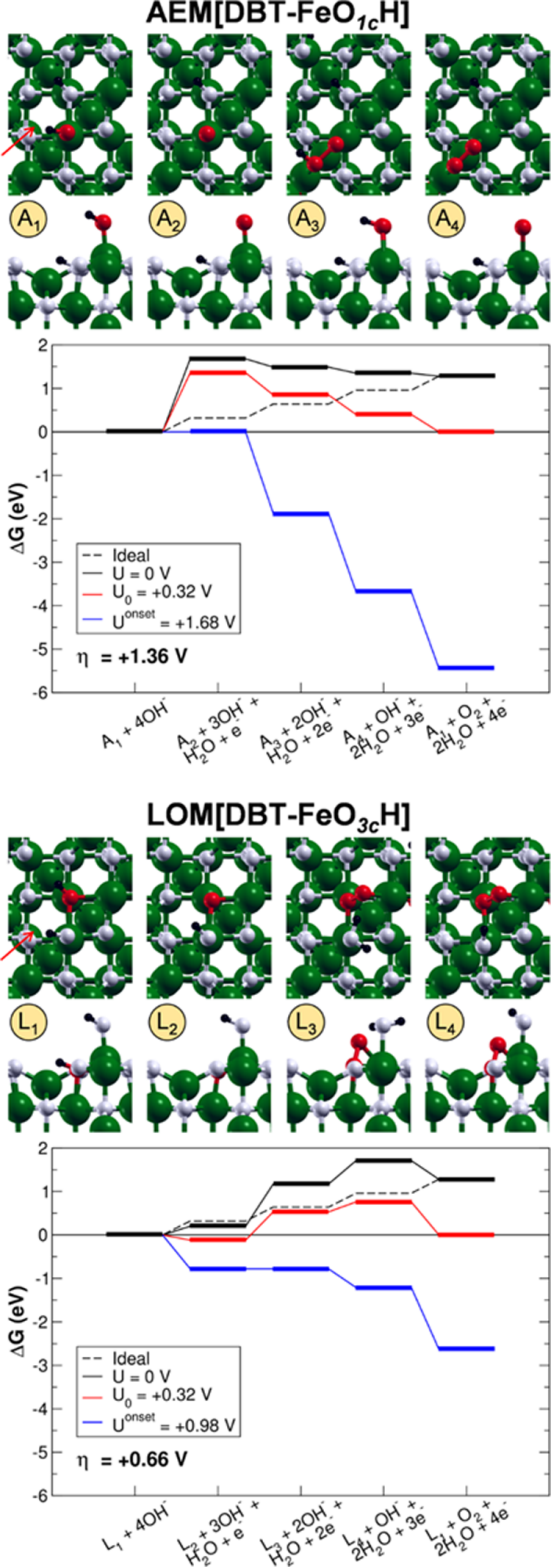
Top (first row) and side (second row) views of the intermediates and energy profiles of the AEM[DBT-FeO_1c_H] and LOM[DBT-FeO_3c_H] OER pathways. The intermediates are labeled as in Figure 2. The green, black, white, and red beads represent Fe, H, O and O involved in the OER intermediates, respectively. The orientation of the crystallographic directions is the same as in Figure 1. The red arrows indicate the direction of the side views.

Two mechanisms were investigated for the OER on a clean DBT surface. The first (namely, AEM[DBT-FeO_1c_H]) involves an OH adsorbed on an exposed fivefold coordinated Fe_Oct_^III^ and proceeds via the conventional AEM, whereas the second (namely, LOM[DBT-FeO_3c_H]) involves an OH formed by the adsorption of a proton on a threefold coordinated surface O atom and proceeds via the LOM. In Figure 3, the structures of the intermediates and the energy profiles of the two reaction paths are shown. In Tables S1 and S2, the Gibbs free energy cost of each step, the overpotential, and the onset potential are listed. In Tables S3 and S4, the lowest-energy spin configurations of the intermediates in terms of difference between the number of α and β electrons are listed.

In the AEM[DBT-FeO_1c_H] mechanism, the first step, i.e., the dehydrogenation of the adsorbed OH (A_1_), consists in the formation of an O^–^ species (A_2_) that is computed to hold a Mulliken spin density value of −0.7 μ_B_ (compatible with one unpaired electron) and a Mulliken charge value that is half that of the hydroxyl O atom. After the addition of a hydroxide ion to the O^–^ species in the second step, a negatively charged hydroperoxo OOH intermediate is formed (A_3_). It interacts also in a bridging fashion with another exposed fivefold coordinated Fe_Oct_^III^ in the surroundings. The OOH oxygen atoms hold no spin density and Mulliken charge values similar to that of the O^–^ species, and the O–O bond length is 1.46 Å, in line with the features of a peroxo species. The third step, i.e., the dehydrogenation of the hydroperoxo, leads to a superoxo OO intermediate, which bridges two exposed fivefold coordinated Fe_Oct_^III^ (A_4_). Each of the OO oxygen atoms has a Mulliken spin density value of −0.4 μ_B_ (compatible with an overall unpaired electron) and a Mulliken charge value, which is half that of the O^–^ species. Furthermore, this third step causes a shortening of the O–O bond length to a value of 1.32 Å, which is suggestive of the transition from peroxo to superoxo. Finally, in the fourth step, the catalyst is regenerated and one gaseous O_2_ molecule is released.

The most energetically demanding step of the overall reaction, i.e., the PDS, is the first step with an overpotential η of 1.36 V. Here, the dehydrogenation of the adsorbed OH leads to the formation of the O^–^ species, which is found to be highly unstable with respect to the other intermediates. Such high instability can be justified by the fact that the O^–^ species in the model is not stabilized by any interaction with the surface, whereas the successive intermediates interact with superficial fivefold coordinated Fe_Oct_^III^. Righi et al.^33^ found the same step to be the PDS but with a smaller overpotential of 0.67 V. This discrepancy could be due to the fact that, in their study, the oxo species is stabilized by the presence of both implicit and explicit aqueous solvents. Moreover, the theoretical description of the Fe^III^–O^–^ pair is a sensitive issue that depends on the adopted computational method (in particular, the percentage of exact exchange in the hybrid functional, as discussed by Righi et al.,^75^ which may affect the energetics of the reaction (see our comparison in Figure S1 in the Supporting Information)).

In the LOM[DBT-FeO_3c_H] mechanism, the first step consists in the dehydrogenation of the OH group formed by the adsorption of the dissociated proton from water on a surface oxygen (L_1_ → L_2_). In this case, no O^–^ species is formed because the electron to be extracted is removed from one Fe^II^ deep in the Fe_3_O_4_ slab model and not from the surface oxygen. The oxidation of one Fe^II^ ion is confirmed by the increase in its Mulliken spin density and charge (from 3.7 to 4.2 μ_B_ and from 1.9 to 2.2, respectively). In the second step, the peroxo OO intermediate is formed inside the magnetite lattice and, in parallel, a proton transfer to the OH on a fivefold coordinated Fe_Oct_^III^ takes place, leading to an adsorbed undissociated water molecule (L_3_). The initial amount of Fe^II^ ions in the surface is restored. The OO oxygen atoms have no significant spin density, and Mulliken charge values are half those of the OH group, with a O–O bond length of 1.47 Å, in line with the features of a peroxo species. We have also investigated a different intermediate structure at this step of reaction, as proposed by Righi et al. in ref (33), where a hydroperoxo OOH species is formed through the binding of a hydroxide ion to a surface oxygen (see Figure S2 in the Supporting Information). This alternative intermediate is, however, much less stable (by +0.60 eV), probably because the surface O atom acquires a fourfold coordination, which is not compatible with a −I oxidation state. As a third step, the adsorbed water molecule in the most stable intermediate in L_3_ is dehydrogenated. Again, a hydroxide ion is formed, and the electron is removed from one Fe^II^ deep in the Fe_3_O_4_ slab model (L_4_). The peroxo OO intermediate is not affected by this reaction, as confirmed by the Mulliken spin density and charge values. Finally, in the fourth step, the catalyst is regenerated and one gaseous O_2_ molecule is released.

The PDS of the LOM reaction path is the second step with an overpotential η of 0.66 V. The formation of the peroxo OO intermediate inside the lattice is reasonably the most energetically demanding step because it implies the intercalation of one oxygen atom in the Fe_3_O_4_ lattice. An overpotential of 0.66 V is in line with the values reported in the computational literature for bare magnetite,^33,38^ as discussed above.

### 4.2 Incorporated Ti, Co, and Ni in the DBT Surface

Incorporated Ti, Co, and Ni atoms (namely, Ti_in_@DBT, Co_in_@DBT, and Ni_in_@DBT, respectively) were studied through the substitution of one Fe_Oct_^III^ ion in the third layer of the DBT surface slab model, as represented in the left panels of Figure 1a. For all metals, different electronic configurations were investigated by varying the overall magnetization of the system. Only the lowest-energy spin configurations are reported and discussed below.

Ti is found to be in the +IV oxidation state (d^0^), in line with the absence of spin density. The substitution of one Fe^III^ with one Ti^IV^ causes the reduction of one neighboring Fe^III^ ion to Fe^II^ in order to keep the charge neutrality of the system (i.e., one Fe^III^–Fe^III^ pair is substituted by one Ti^IV^–Fe^II^ pair). The appearance of an additional Fe^II^ ion in the slab model is confirmed by the reduction of the Mulliken spin density and charge (from 4.2 to 3.7 μ_B_ and from 2.2 to 1.9, respectively) on that Fe site. Another configuration with Ti^III^ (d^1^) is also found. The Ti atom is characterized by a Mulliken spin density of +0.9 μ_B_, indicating the presence of an unpaired electron, and a reduced Mulliken charge with respect to the Ti^IV^ one. Here, none of the Fe^III^ ions is reduced to Fe^II^. However, this alternative spin configuration is higher in energy by ∼0.7 eV; therefore, we did not further consider it in the following for the OER investigation.

Co is found to be in the +II oxidation state (d^7^) in the high-spin configuration, as demonstrated by a Mulliken spin density value of +2.7 μ_B_, compatible with three parallel (spin-up) unpaired electrons. The substitution of one Fe^III^ with one Co^II^ causes the oxidation of one neighboring Fe^II^ ion to Fe^III^ in order to keep the charge neutrality of the system (with the net effect of exchanging one Fe^II^ ion with one Co^II^ ion). The oxidation of one Fe^II^ ion is confirmed by the increase in its Mulliken spin density and charge values (from 3.7 to 4.2 μ_B_ and from 1.9 to 2.2, respectively). Another configuration with low-spin Co^III^ (d^6^) is also found. No significant spin density is observed on the Co atom, whose Mulliken charge is higher with respect to that of Co^II^. None of the Fe ions are involved in any change of the oxidation state. Being the Co^III^ configuration higher in energy by ∼1.3 eV, it will not be further considered in the following for the OER investigation.

Ni is found to be in the +II oxidation state (d^8^) in the high-spin configuration, as demonstrated by a Mulliken spin density value of +1.7 μ_B_, compatible with two parallel (spin-up) unpaired electrons. As in the case of Co^II^, the substitution of one Fe^III^ with one Ni^II^ causes the oxidation of one neighboring Fe^II^ ion to Fe^III^ in order to keep the charge neutrality of the system (with the net effect of exchanging one Fe^II^ ion with one Ni^II^ ion). Another configuration with low-spin Ni^II^ (d^8^), with no significant spin density on the Ni atom, is also found, but it is higher in energy by ∼0.8 eV; thus, it will not be further investigated.

### 4.3 OER on the DBT Surface Incorporating Ti, Co, and Ni

As already done for the clean DBT surface, two mechanisms were investigated for the OER on Ti_in_@DBT, Co_in_@DBT, and Ni_in_@DBT. The first (namely, AEM[Ti_in_@DBT-FeO_1c_H] and AEM[Co_in_@DBT-FeO_1c_H]) involves an OH adsorbed on an exposed fivefold coordinated Fe_Oct_^III^ and proceeds via the conventional AEM, whereas the second (namely, LOM[Ti_in_@DBT-TiO_3c_H], LOM[Ti_in_@DBT-FeO_3c_H], LOM[Co_in_@DBT-CoO_3c_H], and LOM[Ni_in_@DBT-NiO_3c_H]) involves an OH formed by the adsorption of a proton from water dissociation on a surface oxygen and proceeds via the LOM. The LOM[Ti_in_@DBT-TiO_3c_H] and LOM[Ti_in_@DBT-FeO_3c_H] cases differ only in the coordination sphere of the OH, which is the starting point of the reaction: in LOM[Ti_in_@DBT-TiO_3c_H], the OH is directly bonded to the Ti^IV^ ion, whereas in LOM[Ti_in_@DBT-FeO_3c_H], it is only coordinated to Fe ions. In Figure S3 and in Figures S4 and 4, the structures of the intermediates and the energy profiles of the pathways via AEM and via LOM are reported, respectively. In Tables S1 and S2, the Gibbs free energy cost of each step, the overpotential, and the onset potential are listed. In Tables S3 and S4, the lowest-energy spin configurations of the intermediates in terms of difference between the number of α and β electrons and the spin density on the incorporated transition-metal atom are listed.

**Figure 4:**
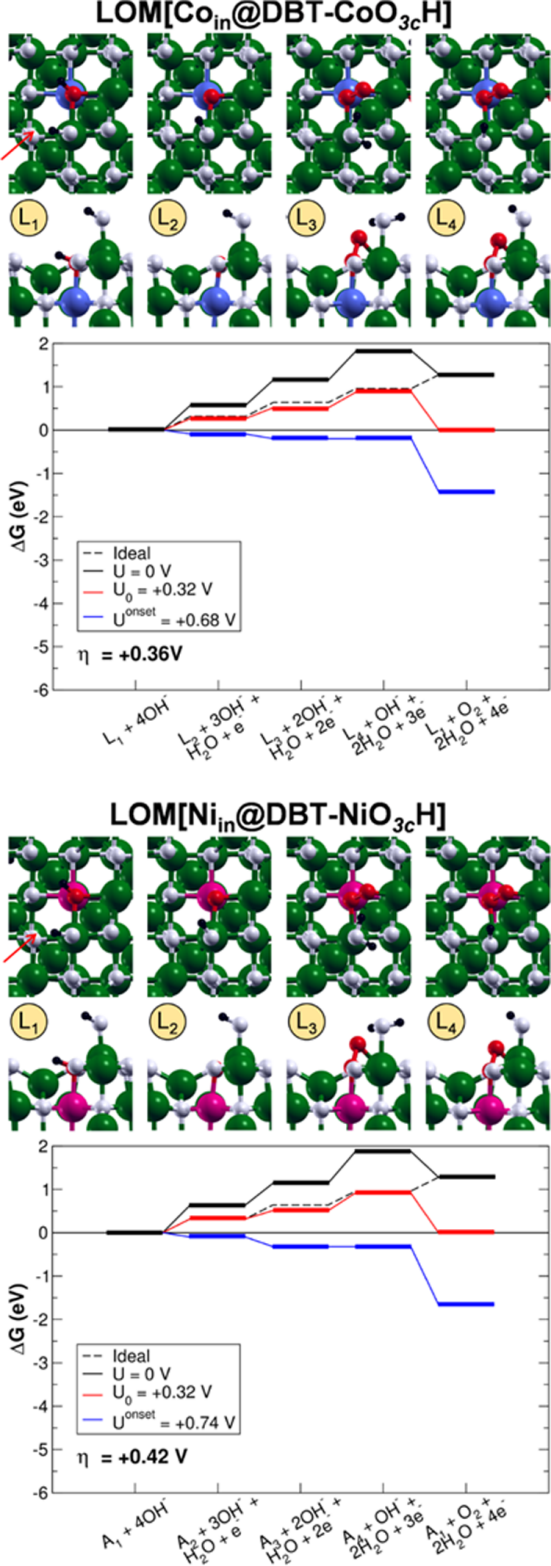
Top (first row) and side (second row) views of the intermediates and energy profiles of the LOM[Co_in_@DBT-CoO_3c_H] and LOM[Ni_in_@DBT-NiO_3c_H] OER pathways. The intermediates are labeled as in Figure 2. The green, black, white, red, blue, and pink beads represent Fe, H, O, O involved in the OER intermediates, Co, and Ni, respectively. The orientation of the crystallographic directions is the same as in Figure 1. The red arrows indicate the direction of the side views.

First, the AEM[Ti_in_@DBT-FeO_1c_H] and AEM[Co_in_@DBT-FeO_1c_H] reaction pathways are analyzed (see Figure S3 in the Supporting Information). The OER is found to proceed through the same intermediates that were characterized above for the clean DBT surface. The formation of the O^–^ species (with Mulliken spin density values of −0.7 μ_B_) is followed by that of the hydroperoxo OOH species (oxygen atoms with no spin density and Mulliken charge values like those of the O^–^ species). The subsequent formation of the superoxo OO through the dehydrogenation of OOH shortens the O–O bond from 1.46 to 1.32 Å. Each of the OO oxygen atoms holds a Mulliken spin density value of −0.4 μ_B_ (AEM[Ti_in_@DBT-FeO_1c_H]) and +0.6 μB (AEM[Co_in_@DBT-FeO_1c_H]), which is compatible with one overall unpaired electron on the superoxo OO group, and a Mulliken charge value that is half that of the O^–^ species. For both AEM[Ti_in_@DBT-FeO_1c_H] and AEM[Co_in_@DBT-FeO_1c_H], the OOH and OO fragments bind in a bridging fashion with exposed fivefold coordinated Fe_Oct_^III^ ions in the surroundings, as observed above for the clean DBT surface.

Also, the energetics seems not to be significantly affected by the presence of the dopants. As in the case of AEM[DBT-FeO_1c_H], the PDS is the first step, i.e., the dehydrogenation of the adsorbed OH, with overpotential values of 1.25 and 1.28 V for AEM[Ti_in_@DBT-FeO_1c_H] and AEM[Co_in_@DBT-FeO_1c_H], respectively, similar to that found for the clean DBT (1.36 V).

Thus, the incorporation of Ti and Co in the third layer of the Fe_3_O_4_(001) surface (Ti_in_@DBT and Co_in_@DBT) does not affect the AEM for the OER, both in the structural or energetic features. On the contrary, when incorporated dopants are directly involved in the OER intermediates, the energetics of the reaction is noticeably changed, as we will detail below.

The LOM[Ti_in_@DBT-TiO_3c_H], LOM[Ti_in_@DBT-FeO_3c_H] (see Figure S4 in the Supporting Information), LOM[Co_in_@DBT-CoO_3c_H], and LOM[Ni_in_@DBT-NiO_3c_H] (see Figure 4) reaction pathways are then analyzed. Again, the OER is found to proceed through the same intermediates as found for the clean DBT surface above. The deprotonation of the lattice OH and the concomitant oxidation of one Fe^II^ are followed by the formation of the peroxo OO species inside the surface lattice. Subsequent proton and electron exchanges lead to the release of one gaseous O_2_ molecule and the regeneration of the catalyst. In all cases, the OO oxygen atoms have no significant spin density, Mulliken charge values are half those of the O atom in the OH group, and the O–O bond length is 1.47 Å, in line with the features of a peroxo species. The mechanism via the formation of the alternative intermediate, a lattice hydroperoxo, has also been evaluated, but again it is found to be very unstable as for the LOM[DBT-FeO_3c_H] case.

If the presence of the dopants does not affect the intermediates and their structure, we cannot state the same regarding the energetics for the reaction profiles in the case of LOM[Ti_in_@DBT-TiO_3c_H], LOM[Co_in_@DBT-CoO_3c_H], and LOM[Ni_in_@DBT-NiO_3c_H]. Only for LOM[Ti_in_@DBT-FeO_3c_H], we observe almost identical results as for the clean surface: the PDS is the formation of the peroxo OO intermediate inside the lattice with an overpotential of 0.64 V (versus 0.66 V for the clean DBT). This is due to the fact that the lattice OH—which is the starting point for the OER—and the other intermediates are coordinated only to Fe ions, as in the case of the clean surface. On the contrary, for LOM[Ti_in_@DBT-TiO_3c_H], the same PDS is characterized by a large increase in the overpotential value up to 1.29 V. From Figure S4, it may seem that this is due to an additional stabilization of the oxo O intermediate in LOM[Ti_in_@DBT-TiO_3c_H], but this is not the actual reason. All of the other intermediates in LOM[Ti_in_@DBT-TiO_3c_H] are destabilized with respect to their equivalent in LOM[Ti_in_@DBT-FeO_3c_H] (compare the two panels in Figure S4), whereas the oxo O one is almost energetically equivalent. Such behavior may suggest that OH and OO intermediates have a lower affinity with Ti than with Fe.

Interestingly, in the cases of LOM[Co_in_@DBT-CoO_3c_H] and LOM[Ni_in_@DBT-NiO_3c_H], the PDS is the third step, which corresponds to the dehydrogenation of the water molecule adsorbed on an exposed fivefold coordinated Fe_Oct_^III^, with an overpotential of 0.36 and 0.42 V, respectively. The promising energy profile associated to this mechanism could be related to the good affinity between OER intermediates and Co and Ni, whose oxophilicity—especially when involved in Fe alloys—is well-known. Indeed, mixed Fe–Co and Fe–Ni oxides are among the most promising materials for OER electrocatalysts.^6,9,11,12,14−17^

### 4.4 Co, Ni, and Cu Adatoms on SCV

Co, Ni, and Cu adatoms (namely, Co_ad_@SCV, Ni_ad_@SCV, and Cu_ad_@SCV, respectively) were studied by binding them with two lattice O atoms of the SCV surface, as represented in the right panels of Figure 1a. Different electronic and spin configurations were investigated by varying the overall magnetization of the system. Only the lowest-energy configurations are reported and discussed in the following.

Similarly to what observed when Co was incorporated in the magnetite lattice in previous sections, the Co adatom preferentially adopts the +II oxidation state (d^7^) in the high-spin configuration, as confirmed by the Mulliken spin density value of +2.5 μ_B_, compatible with the three parallel unpaired electrons with respect to the aligned lattice octahedral Fe ions. For the charge neutrality of the system, the oxidation of the Co adatom to the +II oxidation state must be compensated by the reduction of two Fe^III^ ions, which is confirmed by the reduction of their Mulliken spin density and charge (from 4.2 to 3.7 μ_B_ and from 2.2 to 1.9, respectively). The spin flip of the Co^II^ unpaired electrons from a parallel to an antiparallel spin configuration with respect to the aligned lattice octahedral Fe ions (Mulliken spin density value from +2.5 to −2.6 μ_B_) is found to be slightly unfavored by ∼0.1 eV. We notice that water dissociation on the surface inverts this tendency by stabilizing the antiparallel configuration, which will be considered when we computed the reaction energy profile for OER in the next section. We could also localize the high- and low-spin configurations for Co^I^ (d^8^), but they are unfavored by ∼0.2 and ∼1.2 eV, respectively.

Differently from Co, the Ni adatom prefers the +I oxidation state (3d^8^4s^1^) in the low-spin configuration, as confirmed by the Mulliken spin density value of −1.0 μ_B_, compatible with one antiparallel unpaired electron with respect to the aligned lattice octahedral Fe ions. Notice that the parallel configuration is ∼0.1 eV higher in energy. The presence of the adatom in a +I oxidation state implies the reduction of one Fe^III^ ion, which is confirmed by a lower Mulliken spin density and charge. A high-spin Ni^II^ (d^8^) configuration (with two antiparallel unpaired electrons and Mulliken spin density of −1.6 μ_B_) is also found, although it is unfavored by ∼0.2 eV. However, after water dissociation on the surface (see Figure 1b), only Ni^II^ species could be localized, and thus, it will be used in the next section as a starting point for the OER reaction paths.

Like Ni, Cu prefers the +I oxidation state (d^10^), as proved by the absence of significant spin density. One Fe^III^ ion in the slab model becomes reduced to Fe^II^ in order to keep the charge neutrality of the system. After heterolytic water dissociation, the Cu is further oxidized to the +II oxidation state and one more Fe^III^ ion in the slab model is reduced. This species, i.e., Cu^II^ in d^9^ configuration with one antiparallel unpaired electron with respect to the aligned lattice octahedral Fe ions (Mulliken spin density of −0.6 μ_B_), will be further considered as the starting point of the OER investigation.

As mentioned in Section 1, the stability is a key factor to be considered in the design of potential new Fe_3_O_4_(001)-based electrocatalysts. For this reason, we investigated the stability of the metal atoms deposited as adatoms on the SCV surface (namely, Co_ad_@SCV, Ni_ad_@SCV, and Cu_ad_@SCV) against dissolution under electrochemical OER conditions, as detailed in the Supporting Information (see Figure S5 and Section S1 in the Supporting Information). Co_ad_@SCV and Ni_ad_@SCV are stable against dissolution under the application of the potential at which OER is experimentally observed to take place at the magnetite surface, whereas Cu_ad_@SCV is not.

### 4.5 OER on Co, Ni, and Cu Adatoms on SCV

**Figure 5:**
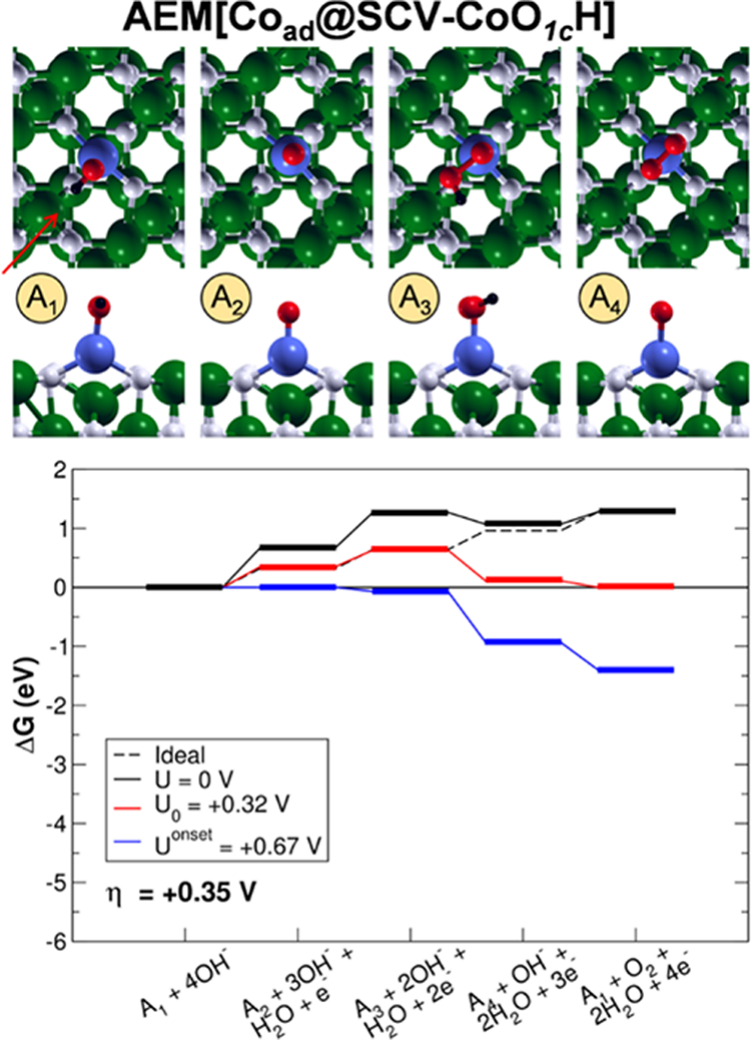
Top (first row) and side (second row) views of the intermediates and energy profiles of the AEM[Co_ad_@SCV-CoO_1c_H] OER pathway. The intermediates are labeled as in Figure 2. The green, black, white, red, and blue beads represent Fe, H, O, O involved in the OER intermediates, and Co, respectively. The orientation of the crystallographic directions is the same as in Figure 1. The red arrow indicates the direction of the side views.

**Figure 6:**
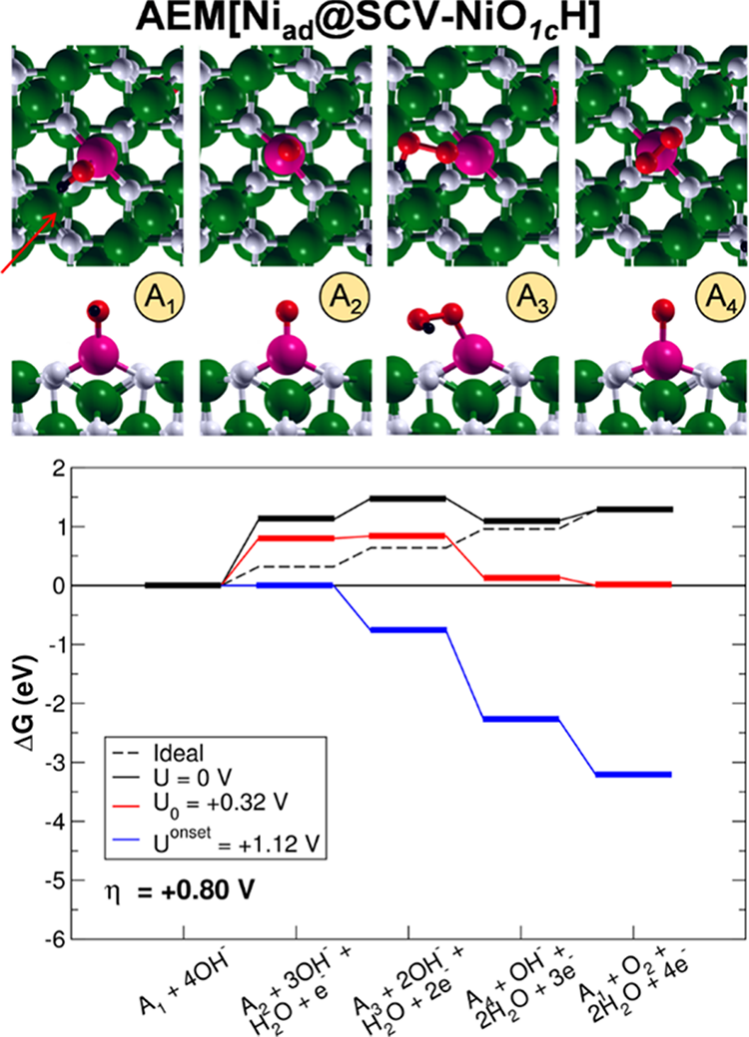
Top (first row) and side (second row) views of the intermediates and energy profiles of the AEM[Ni_ad_@SCV-NiO_1c_H] OER pathway. The intermediates are labeled as in Figure 2. The green, black, white, red, and pink beads represent Fe, H, O, O involved in the OER intermediates, and Ni, respectively. The orientation of the crystallographic directions is the same as in Figure 1. The red arrow indicates the direction of the side views.

**Figure 7:**
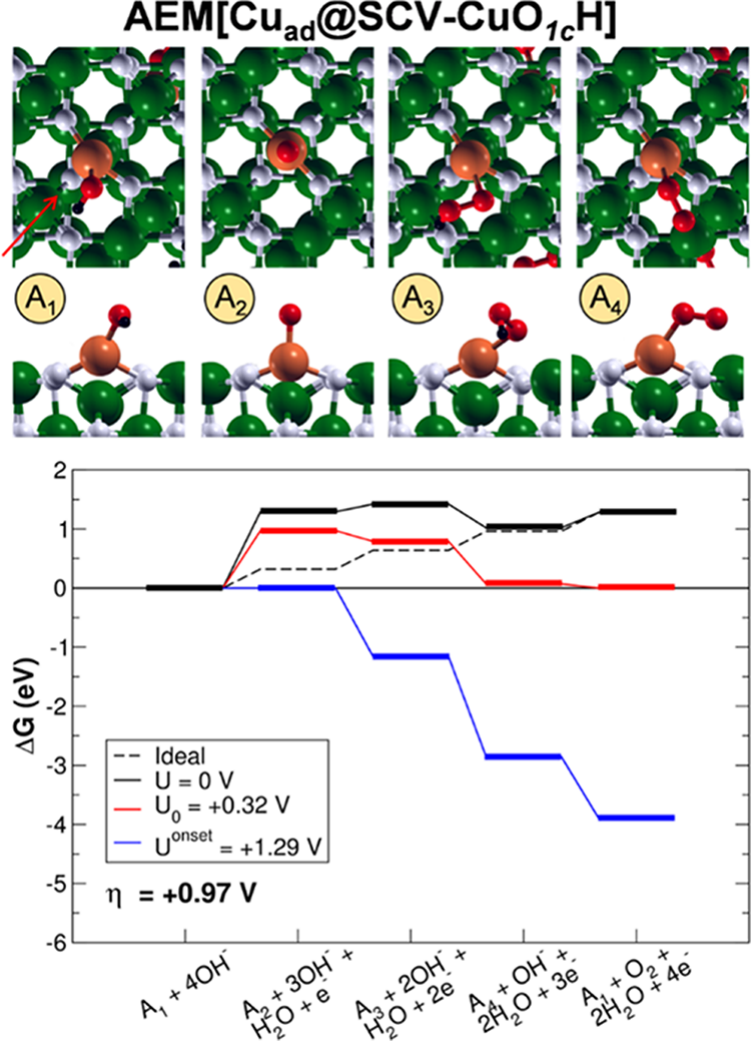
Top (first row) and side (second row) views of the intermediates and energy profiles of the AEM[Cu_ad_@SCV-CuO_1c_H] OER pathway. The intermediates are labeled as in Figure 2. The green, black, white, red, and brown beads represent Fe, H, O, O involved in the OER intermediates, and Cu respectively. The orientation of the crystallographic directions is the same as in Figure 1. The red arrow indicates the direction of the side views.

For the OER on Co_ad_@SCV, Ni_ad_@SCV, and Cu_ad_@SCV, only the conventional AEM mechanism was investigated (namely, AEM[Co_ad_@SCV-CoO_1c_H], AEM[Ni_ad_@SCV-NiO_1c_H], and AEM[Cu_ad_@SCV-CuO_1c_H]), starting from an OH species adsorbed on the metal adatoms. In Figures 5–7, the structures of the intermediates and the energy profiles of these OER pathways are reported. In Table S1, the Gibbs free energy cost of each step, the overpotential, and the onset potential are listed. In Tables S3 and S4, the lowest-energy spin configurations of the intermediates in terms of difference between the number of α and β electrons and the spin density on the transition-metal adatom are listed. The LOM mechanism was not considered for these systems because the OER intermediates in LOM would not directly involve the metal adatoms. As a consequence of this, it is reasonable to expect similar results to those found for the clean surface (LOM[DBT-FeO_3c_H]), as also observed for AEM[Co_in_@DBT-FeO_1c_H], AEM[Ti_in_@DBT-FeO_1c_H], and LOM[Ti_in_@DBT-FeO_3c_H], whose reaction intermediates did not directly interact with the dopants and whose results were almost identical to those of the pristine surface (AEM[DBT-FeO_1c_H] and LOM[DBT-FeO_3c_H], respectively).

The OER intermediates via AEM[Co_ad_@SCV-CoO_1c_H] (see Figure 5), AEM[Ni_ad_@SCV-NiO_1c_H] (Figure 6), and AEM[Cu_ad_@SCV-CuO_1c_H] (see Figure 7) pathways are the same as for the DBT-based surface models, i.e., AEM[DBT-FeO_1c_H], AEM[Ti_in_@DBT-FeO_1c_H], and AEM[Co_in_@DBT-FeO_1c_H]. The O^–^ species, formed by the dehydrogenation of the OH group on the metal adatom, is attacked by a hydroxide ion to generate the hydroperoxo OOH fragment. The O^–^ species are characterized by Mulliken spin densities of −1.1/–1.2 μ_B_, whereas the OOH oxygen atoms have no significant Mulliken spin density and Mulliken charges similar to those of the O^–^ species. In the AEM[Ni_ad_@SCV-NiO_1c_H] and AEM[Cu_ad_@SCV-CuO_1c_H] cases, the OOH fragment interacts through a weak hydrogen bond (bond length of 1.8 Å and bond angle of 130 and 145°, respectively) with a surface O atom, whereas for AEM[Co_ad_@SCV-CoO_1c_H], it does not. As a third step, the dehydrogenation of the hydroperoxo OOH leads to the superoxo OO species with a shortening the O–O bond length from 1.48, 1.46, and 1.43 to 1.35, 1.32, and 1.30 Å, for AEM[Co_ad_@SCV-CoO_1c_H], AEM[Ni_ad_@SCV-NiO_1c_H], and AEM[Cu_ad_@SCV-CuO_1c_H], respectively. In the case of Co and Ni, a side-on superoxo species is formed: the unpaired electron is equally shared between the two oxygen atoms, as confirmed by the Mulliken spin densities of −0.6 μ_B_ for each of them. In the case of Cu, an end-on superoxo species is formed: the unpaired electron is more localized on the oxygen atom bonded to the Cu adatom (Mulliken spin density of −0.7 versus −0.5 μ_B_ for the terminal oxygen atom).

Although the reaction intermediates are the same as on the pristine surface, the energetics is largely affected by the presence of the adatoms. The PDS is still identified for all of the systems with the O^–^ formation, but the overpotential is reduced with respect to the other systems investigated in the previous section (namely, AEM[DBT-FeO_1c_H], AEM[Ti_in_@DBT-FeO_1c_H], and AEM[Co_in_@DBT-FeO_1c_H)]. In particular, η values are 0.35, 0.80, and 0.97 eV for AEM[Co_ad_@SCV-CoO_1c_H], AEM[Ni_ad_@SCV-NiO_1c_H], and AEM[Cu_ad_@SCV-CuO_1c_H], respectively. Despite the fact that mixed Ni/Fe and Co/Fe oxides were both reported to be excellent OER electrocatalysts,^6,9,11,12,14−17^ here, only Co adatoms are found to improve significantly the catalytic performance of magnetite toward OER with respect to the clean surface. Ni adatoms present high overpotentials, in agreement with recent experimental findings.^50^

As in the case of OER via LOM (LOM[Co_in_@DBT-CoO_3c_H]), the Co-doped system is the best performing also via AEM (AEM[Co_ad_@SCV-CoO_1c_H]). These results could be due to the good affinity (neither too strongly nor too weakly bound) between the OER intermediates and Co. To give ground to this thesis, adsorption energies (*E*_Ads_) were computed for the dissociative adsorption of one water molecule on Co_ad_@SCV, Ni_ad_@SCV, and Cu_ad_@SCV. The adsorption of OH on Co is more favored than on Ni and Cu (−0.79 versus −0.58 eV, respectively), in line with the statement above.

Furthermore, the O^–^ species—whose formation corresponds to the PDS—is more stable on the Co adatom rather than on Ni and Cu adatoms, while the energetics of adsorption of the other intermediates is similar on all three adatoms, as suggested by the OER profiles shown in Figures 5–7. To understand this trend, the electronic structures of the species under investigation were computed and analyzed. In Figure S6 in the Supporting Information, the PDOS of the OH and O intermediates for the AEM[Co_ad_@SCV-CoO_1c_H], AEM[Ni_ad_@SCV-NiO_1c_H], and AEM[Cu_ad_@SCV-CuO_1c_H] pathways are shown, where the cyan curves represent the 2p states of the oxygen of the adsorbed intermediates (originated from the dissociative adsorption of one water molecule). These states are partially left empty in the α channel when OH is dehydrogenated to form the O^–^ species, as proven by the appearance of 2p states in the conduction band. The 2p states of the hydroxyl O atom are closer to the Fermi energy for Co_ad_@SVC than Ni_ad_@SCV and Cu_ad_@SCV, as confirmed by the computed 2p-band COM values of −4.2, −4.7, and −4.9 eV, respectively. These descriptors indicate that the 2p states are more easily depleted, leading to a more stable subsequent O^–^ intermediate, when the OH species is adsorbed on the Co adatom.

## 5 Conclusions

In this hybrid DFT study, we performed a comparative investigation of the OER on clean and metal-doped Fe_3_O_4_(001) model electrocatalysts.

For the clean Fe_3_O_4_(001) DBT surface, two OER mechanisms were investigated: the LOM and the conventional AEM. As regards the pathway based on the LOM (i.e., involving a surface oxygen atom of the magnetite lattice), the OER proceeds through the intermediates proposed for the NiFe_2_O_4_(001) surface by Li and Selloni,^38^ giving an overpotential value of 0.66 V, which is similar to that computed by Righi et al.,^33^ even if for a slightly different pathway. As regards the pathway based on the AEM (i.e., involving the oxygen of the adsorbed hydroxide), a high overpotential value of 1.36 V is found due to the highly unstable oxo O^–^ species.

The metal-doped Fe_3_O_4_(001) systems were investigated considering both (i) metal atom incorporation in a subsurface vacancy in the third layer (Ti, Co, and Ni) or (ii) metal deposition as adatoms on the surface (Co, Ni, and Cu). When metal atoms are incorporated in the lattice, results are similar to those observed for the clean surface, except when a direct bond between the reaction intermediates and metal is established. Indeed, conventional AEM-based OER pathways on Ti_in_@DBT and Co_in_@DBT give almost identical results to those for the clean DBT. On the contrary, LOM-based OER results on the same systems are affected by the dopants: Ti-incorporated magnetite presents an increased overpotential (η = 1.39 V), whereas Co- and Ni-incorporated reduced ones (η = 0.36 and 0.42 V) compared with the clean magnetite surface (η = 0.66 V). In the case of metal adatoms deposited on the surface, the Co-loaded system is found to be the most promising for the OER with a reasonably low overpotential (η = 0.35 V), whereas Ni and Cu do not improve the performance with respect to the clean surface (η = 0.80 and 0.97 V, respectively).

**Figure 8:**
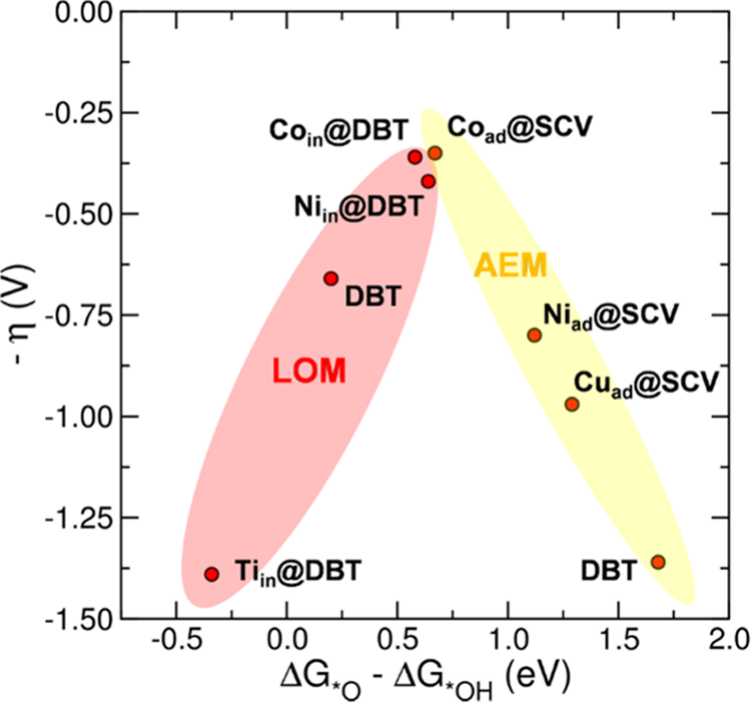
Activity trends toward OER plotted for pristine Fe_3_O_4_(001) and Fe_3_O_4_(001)-supported SACs. The negative value of the theoretical overpotential (−η) is plotted against the Gibbs free energy cost of the hydroxyl deprotonation (ΔG_*O_ – ΔG_*OH_) step. The red area collects data for LOM, whereas the yellow area collects data for AEM. DBT appears in both for direct comparison of SAC activity with the pristine surface.

The most relevant results are displayed in a volcano plot, which is shown in Figure 8. On the top of the volcano, the Co-doped systems present the lowest computed overpotentials (η), followed by the Ni-incorporated one. The right branch of the plot is constituted by the systems where the OER proceeds via AEM, for which the PDS is the deprotonation of the hydroxyl group on the metal adatom (A_1_ → A_2_). The left branch of the plot is constituted by the systems where the OER proceeds via LOM, for which the PDS is a subsequent step (L_2_ → L_3_ or L_3_ → L_4_).

To summarize, transition-metal atoms affect the electrocatalytic performance of magnetite only when they are directly involved in the formation of intermediates through chemical bonds, i.e., metal dopants do not exercise any significant proximity or long-range effects able to affect the energetics of the OER. In particular, Co-doped systems (Co_ad_@SCV and Co_in_@DBT), independent of whether the Co is incorporated in or deposited on the surface, are found to be the most promising electrocatalysts among those investigated in this study due to the well-balanced affinity between the transition-metal atom and the OER intermediates, leading to a decrease in the OER overpotential value of 0.3 V with respect to the clean magnetite surface. Similar results are obtained for Ni-doped systems but only in the case of the incorporated metal.

To conclude, the large set of data reported in this study has proven that both transition-metal atoms loading on or incorporating in the magnetite surface could be successful strategies to improve its electrocatalytic activity for the OER. However, the doping approach should involve the surface layers because the incorporated metal atoms must take part in the formation of the intermediates. The good performance of incorporated surface Co or Ni dopants agrees with the generally recognized high efficiency of mixed Fe oxides as OER catalysts (experimental range: 0.2 V < η < 0.5 V^6,9,11,12,14−17^). In particular, the 0.42 V overpotential value for incorporated Ni is extremely close to the values of 0.42 and 0.44 V that were computed with DFT + *U* methods for the NiFe_2_O_4_(001) surface.^38,68^ In the case of Co-doped systems, the overpotential values for the incorporated in or deposited on species (0.36 and 0.35 V, respectively) are very close to the value of 0.38 V that was computed for the CoFe_2_O_4_(001) surface.^68^ These new results prove that the same performance toward OER can be obtained by introducing a limited quantity of Co or Ni on the Fe_3_O_4_ surface rather than by using Co/Fe or Ni/Fe mixed oxides and, thus, provide experimentalists a clear and practical way to cut the catalyst cost.

## Supporting Information Available

Energy profiles of the AEM[DBT-FeO_1c_H] OER pathway calculated at different percentage of exact exchange; unfavored hydroperoxo OOH intermediate of the LOM[DBT-FeO_3c_H] OER pathway; intermediates and energy profiles of the AEM[Ti_in_@DBT-FeO_1c_H], AEM[Co_in_@DBT-FeO_1c_H], LOM[Ti_in_@DBT-TiO_3c_H], and LOM[Ti_in_@DBT-FeO_3c_H] OER pathways; stability of metal adatoms on the SCV surface in terms of dissolution potentials and formation energies; PDOS of the OH and O intermediates on Co_ad_@SVC, Ni_ad_@SCV, and Cu_ad_@SCV; and further energetic and magnetic information for the OER pathways (PDF)
