# Supplementary material for: Improving the Oxygen Evolution Reaction on Fe3O4(001) with Single-Atom Catalysts
Source: ACS Catal. 2023 Mar 24;13(7):4811–23. doi: 10.1021/acscatal.3c00337 (PMC10088028; doi:10.1021/acscatal.3c00337)
Supplement: Supplementary file 1 — cs3c00337_si_001.pdf [file cs3c00337_si_001.pdf]

Supporting Information

Improving Oxygen Evolution Reaction on Fe_3O_4(001) with Single-Atom Catalysts

Enrico Bianchetti[1], Daniele Perilli[1], and Cristiana Di Valentin[1,2,*]

[1] Dipartimento di Scienza dei Materiali, Università di Milano Bicocca,

Via Roberto Cozzi 55, 20125 Milano, Italy

[2] BioNanoMedicine Center NANOMIB, Università di Milano Bicocca,

Via Raoul Follereau 3, 20900 Monza, Italy

[*] Corresponding author: cristiana.divalentin@unimib.it

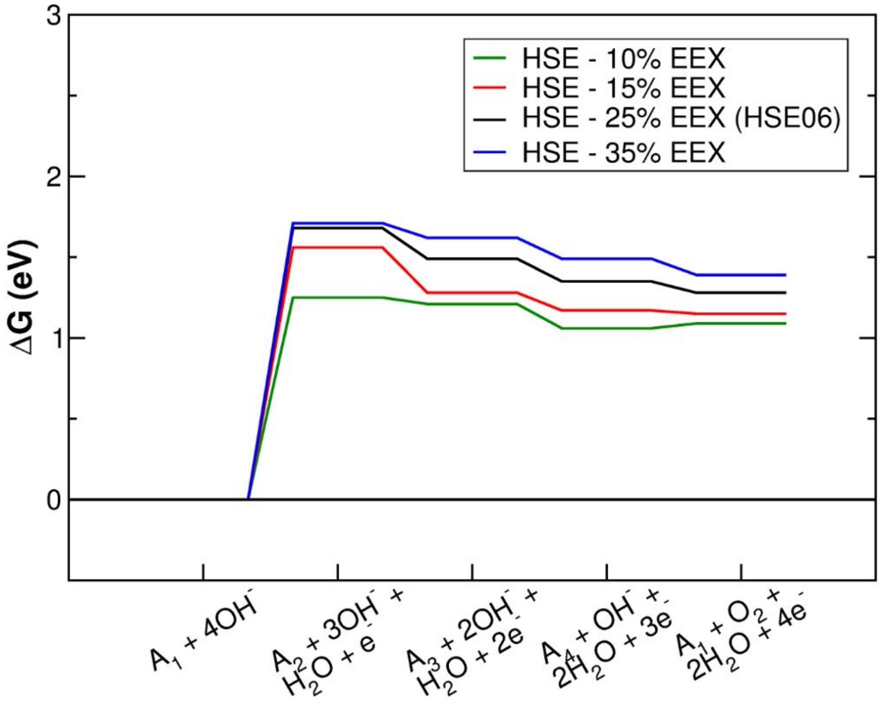

Figure S1. Energy profiles of the AEM[DBT-FeO1cH] OER pathway calculated at different percentage of Exact Exchange (EEX). The intermediates are labeled as in Figure 2 in the main text and their structures are kept fixed with respect to those computed at the HSE06 level (black profile). By reducing the percentage of EEX to 10% (green profile), the O oxo intermediate (A_2) is further stabilized by ~0.15 eV with respect to the other intermediates (A_3 and A_4).

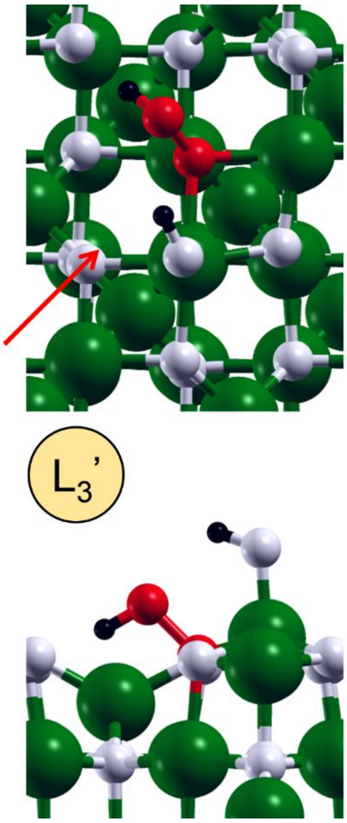

Figure S2. Top and side view of the hydroperoxo OOH intermediate as proposed by Righi et al.[1] for the LOM[DBT-$FeO_{3c}H$] OER pathway. The intermediate is labeled according to Figure 2. The green, black, white, and red beads represent Fe, H, O and O involved in the OER intermediates, respectively. The orientation of the crystallographic directions is the same as in Figure 1. The red arrow indicates the direction of the side view.

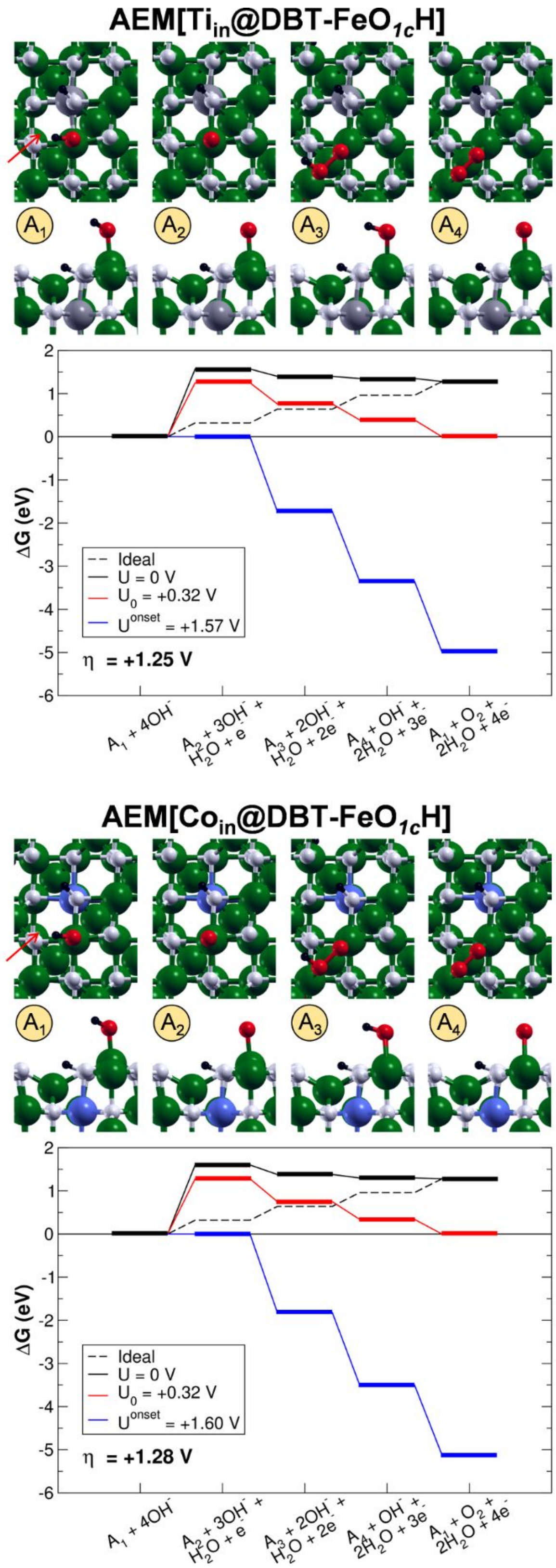

Figure S3. Top (first row) and side (second row) views of the intermediates and energy profiles of the AEM[Ti_{in}@DBT-FeO_{1c}H] and AEM[Co_{in}@DBT-FeO_{1c}H] OER pathways. The intermediates are labeled as in Figure 2. The green, black, white, red, grey, and blue beads represent Fe, H, O, O involved in the OER intermediates, Ti, and Co respectively. The orientation of the crystallographic directions is the same as in Figure 1. The red arrows indicate the direction of the side views.

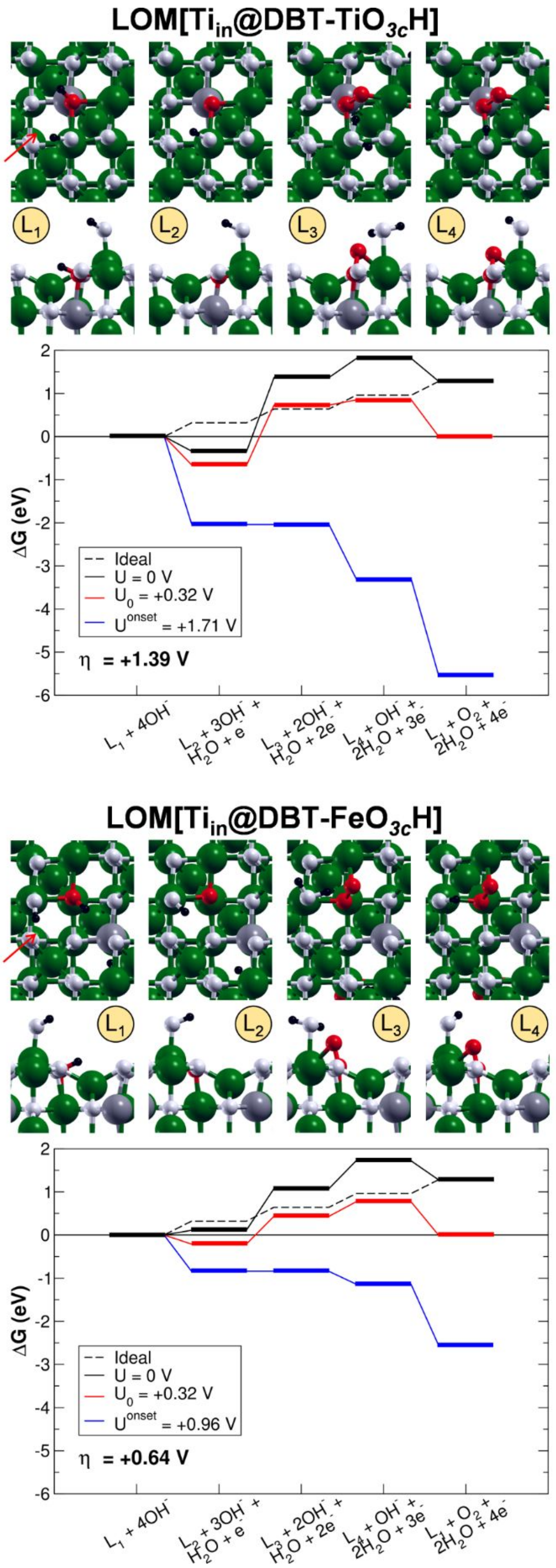

Figure S4. Top (first row) and side (second row) views of the intermediates and energy profiles of the LOM[Ti$_{in}$@DBT-TiO$_{3c}$H] and LOM[Ti$_{in}$@DBT-FeO$_{3c}$H] OER pathways. The intermediates are labeled as in Figure 2. The green, black, white, red, and grey beads represent Fe, H, O, O involved in the OER intermediates, and Ti, respectively. The orientation of the crystallographic directions is the same as in Figure 1. The red arrows indicate the direction of the side views.

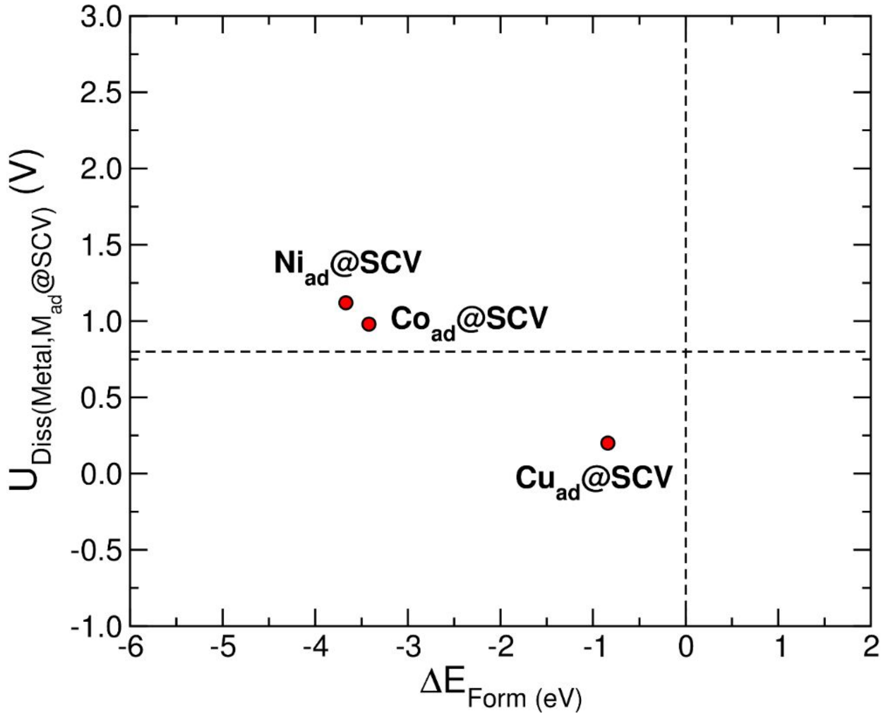

Figure S5. Dissolution potentials (with respect to SHE at pH=14) versus formation energies of metal atoms deposited as adatoms on the SCV Fe_3O_4(001) surface. The horizontal dashed line represents the potential at which OER is experimentally taking place at the magnetite surface (0.8 V with respect to SHE at pH=14[2,3]). The vertical dashed line represents $\Delta E_{Form} = 0$ eV, which is the upper bound to the stability of the M_{ad}@SCV systems. M_{ad}@SCV catalysts in the upper left quadrant (ΔE_{Form} < 0 eV and $U_{Diss(Metal,M_{ad}@SCV)}$ > 0.8 V) are promising in terms of the stability. The ΔE_{Form} and $U_{Diss(Metal,M_{ad}@SCV)}$ values are listed in Table S5. See Section S1 below for further details about the calculation of dissolution potentials and formation energies.

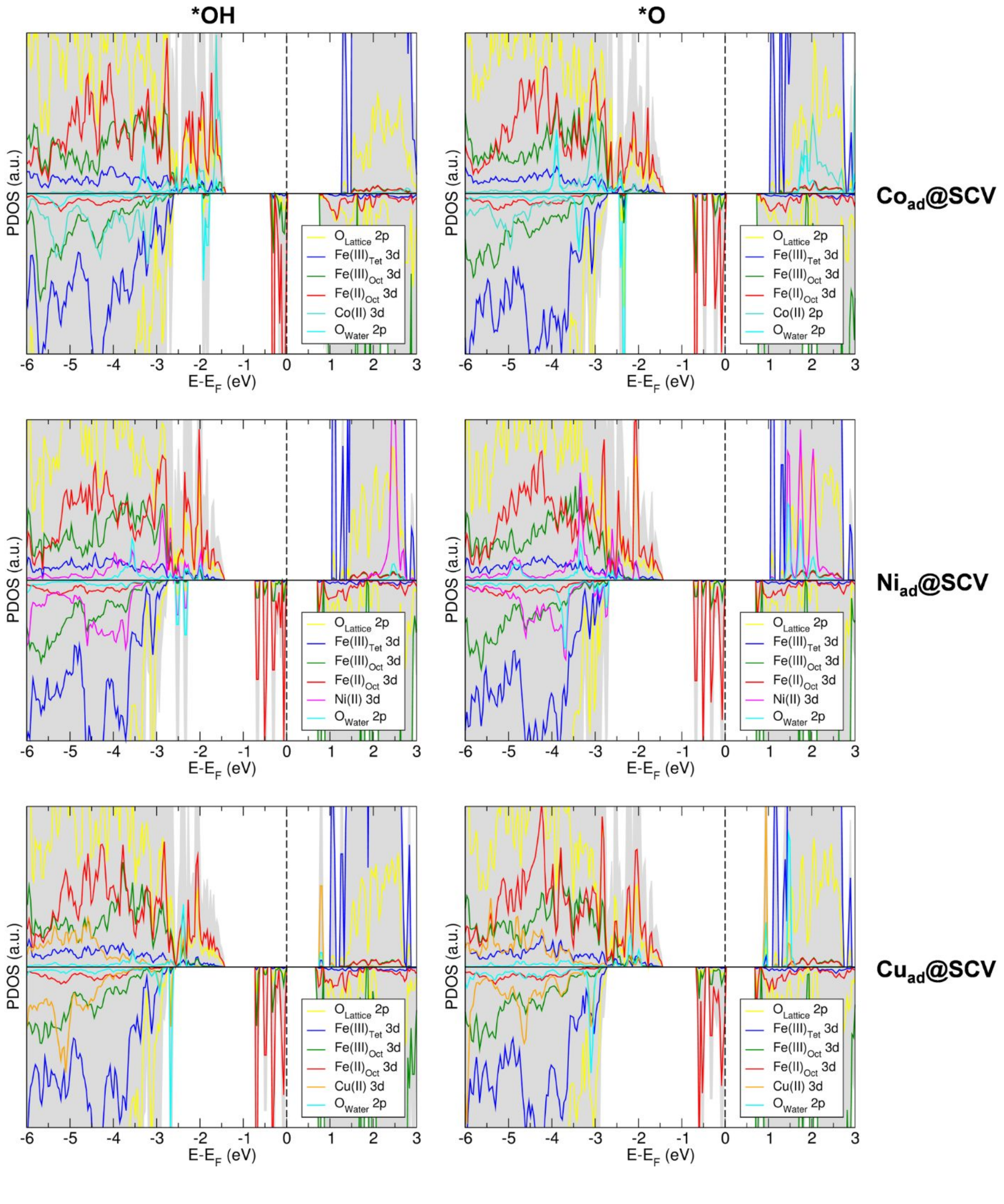

Figure S6. PDOS of the OH and O intermediates on the Co$_{ad}$@SVC, Ni$_{ad}$@SCV and Cu$_{ad}$@SCV model electrocatalysts.

Table S1. Gibbs free energy cost (in eV) of the four PCET steps, overpotential (in V) and onset potential (in V) for the OER pathways via AEM.

	**AEM**					
	DBT-$FeO_{1c}H$	Ti_{in}@DBT-$FeO_{1c}H$	Co_{in}@DBT-$FeO_{1c}H$	Co_{ad}@SCV-$CoO_{1c}H$	Ni_{ad}@SCV-$NiO_{1c}H$	Cu_{ad}@SCV-$CuO_{1c}H$
Spin ($M_{in/ad}$)	-	-0.2	+2.7	-2.6	-1.6	-0.6
$\Delta G_{A_1 \to A_2}$	+1.68	+1.57	+1.60	+0.67	+1.12	+1.29
$\Delta G_{A_2 \to A_3}$	-0.19	-0.17	-0.22	+0.60	+0.36	+0.13
$\Delta G_{A_3 \to A_4}$	-0.14	-0.15	-0.08	-0.20	-0.38	-0.40
$\Delta G_{A_4 \to A_1}$	-0.08	-0.08	-0.03	+0.25	+0.17	+0.25
η	+1.36	+1.25	+1.28	+0.35	+0.80	+0.97
U^{onset}	+1.68	+1.57	+1.60	+0.67	+1.12	+1.29

Table S2. Gibbs free energy cost (in eV) of the four PCET steps, overpotential (in V) and onset potential (in V) for the OER pathways via LOM.

	**LOM**				
	DBT-$FeO_{3c}H$	Ti_{in}@DBT-$FeO_{3c}H$	Ti_{in}@DBT-$TiO_{3c}H$	Co_{in}@DBT-$CoO_{3c}H$	Ni_{in}@DBT-$NiO_{3c}H$
Spin ($M_{in/ad}$)	-	-0.2	-0.2	+2.7	+1.7
$\Delta G_{L_1 \to L_2}$	+0.20	+0.11	-0.34	+0.58	+0.64
$\Delta G_{L_2 \to L_3}$	+0.97	+0.96	+1.71	+0.58	+0.50
$\Delta G_{L_3 \to L_4}$	+0.54	+0.65	+0.44	+0.67	+0.74
$\Delta G_{L_4 \to L_1}$	-0.44	-0.45	-0.54	-0.56	-0.61
η	+0.66	+0.64	+1.39	+0.36	+0.42
U^{onset}	+0.98	+0.96	+1.71	+0.68	+0.74

Table S3. Lowest-energy spin configurations of the four intermediates in terms of difference between the number of α and β electrons (per unit cell) and spin density (in μ_B) on the transition metal atom incorporated (M_{in}) in or deposited as adatom (M_{ad}) on the magnetite surface for the OER pathways via AEM.

	AEM					
	DBT-FeO$_{1c}$H	Ti$_{in}$@DBT-FeO$_{1c}$H	Co$_{in}$@DBT-FeO$_{1c}$H	Co$_{ad}$@SCV-CoO$_{1c}$H	Ni$_{ad}$@SCV-NiO$_{1c}$H	Cu$_{ad}$@SCV-CuO$_{1c}$H
$n_{e_\alpha^-} - n_{e_\beta^-}$ (A_1)	88	76	86	54	56	58
$n_{e_\alpha^-} - n_{e_\beta^-}$ (A_2)	86	74	84	52	54	56
$n_{e_\alpha^-} - n_{e_\beta^-}$ (A_3)	88	76	86	54	56	58
$n_{e_\alpha^-} - n_{e_\beta^-}$ (A_4)	86	74	88	52	54	56
Spin ($M_{in/ad}$)	-	-0.2	+2.7	-2.6	-1.6	-0.6

Table S4. Lowest-energy spin configurations of the four intermediates in terms of difference between the number of α and β electrons (per unit cell) and spin density (in μ_B) on the transition metal atom incorporated (M_{in}) in or deposited as adatom (M_{ad}) on the magnetite surface for the OER pathways via LOM.

	LOM				
	DBT-FeO$_{3c}$H	Ti$_{in}$@DBT-FeO$_{3c}$H	Ti$_{in}$@DBT-TiO$_{3c}$H	Co$_{in}$@DBT-CoO$_{3c}$H	Ni$_{in}$@DBT-NiO$_{3c}$H
$n_{e_\alpha^-} - n_{e_\beta^-}$ (L_1)	88	76	76	86	84
$n_{e_\alpha^-} - n_{e_\beta^-}$ (L_2)	90	78	78	88	86
$n_{e_\alpha^-} - n_{e_\beta^-}$ (L_3)	88	76	76	86	84
$n_{e_\alpha^-} - n_{e_\beta^-}$ (L_4)	90	78	78	88	86
Spin ($M_{in/ad}$)	-	-0.2	-0.2	+2.7	+1.7

S1. Stability against dissolution and formation energy.

To examine the stability of M_{ad}@SCV catalysts under electrochemical OER conditions, we investigated their formation energies as well as their dissolution potentials, exploiting a protocol previously used in literature.[4]

The formation energies for the M_{ad}@SCV catalysts are calculated as

$$\Delta E_{Form} = E_{M_{ad}@SCV} - E_{SCV} - E_{Metal,Bulk}$$

where $E_{M_{ad}@SCV}$, E_{SCV}, and $E_{Metal,Bulk}$ are electronic energies of SCV-supported metal adatom, clean SCV surface, and metal atom in its bulk form, respectively.

In the following, the dissolution reactions and the tabulated reduction potentials (with respect to SHE at pH=14) are reported for the investigated metals, where $M_{(s)}$ is referred to the metal in its bulk form.

$$Co: \quad Co(OH)_2 + 2e^- \rightarrow Co_{(s)} + 2OH^- \qquad U^0 = -0.73\ V$$

$$Ni: \quad Ni(OH)_2 + 2e^- \rightarrow Ni_{(s)} + 2OH^- \qquad U^0 = -0.72\ V$$

$$Cu: \quad Cu(OH)_2 + 2e^- \rightarrow Cu_{(s)} + 2OH^- \qquad U^0 = -0.22\ V$$

We calculated the dissolution potentials of bulk metals ($U^0_{Diss(Metal,Bulk)}$) using the equation: $U^0_{Diss(Metal,Bulk)} = U^0$. If the applied potential U is higher than $U^0_{Diss(Metal,Bulk)}$, the bulk metal atom dissolves in the water environment. However, when the metal atom is loaded as adatom on the SCV surface (M_{ad}@SCV), a stabilization effect may occur. To consider this effect, the dissolution potential for the SCV-supported metal atom ($U_{Diss(Metal,M_{ad}@SCV)}$) is calculated as

$$U_{Diss(Metal,M_{ad}@SCV)} = U^0_{Diss(Metal,Bulk)} - \frac{\mu_{Metal,M_{ad}@SCV} - \mu^0_{Metal,Bulk}}{ne}$$

$$= U^0_{Diss(Metal,Bulk)} - \frac{\Delta E_{Form}}{ne}$$

where $\mu_{Metal,M_{ad}@SCV}$, $\mu^0_{Metal,Bulk}$, n, and e are the chemical potential of metal adatom on the SCV surface, that of bulk metal, the number of electrons involved in the dissolution, and the elementary

charge, respectively. The $\mu_{Metal,M_{ad}@SCV}$ and $\mu^{0}_{Metal,Bulk}$ terms are calculated as $E_{M_{ad}@SCV} - E_{SCV}$ and $E_{Metal,Bulk}$, respectively.

In Figure S5, dissolution potentials of M_{ad}@SCV metals are plotted versus their formation energies. The horizontal dashed line represents the potential at which OER is experimentally taking place at the magnetite surface (0.8 V with respect to SHE at pH=14[2,3]). The vertical dashed line represents $\Delta E_{Form} = 0$ eV, which is the upper bound to the stability of the M_{ad}@SCV systems. Figure S4 combines the two stability criteria, i.e., formation of metal adatom on magnetite surface as well as stability under electrochemical OER conditions. The left side of the vertical dashed line indicates a favorable formation of M_{ad}@SCV catalyst, while the upper side of the horizontal dashed line indicates its stability under electrochemical OER conditions (0.8 V with respect to SHE at pH=14, where the current densities begin to be measured[2,3]). Therefore, M_{ad}@SCV catalysts in the upper left quadrant (ΔE_{Form} < 0 eV and $U_{Diss(Metal,M_{ad}@SCV)}$ > 0.8 V) are promising in terms of the stability. The ΔE_{Form} and $U_{Diss(Metal,M_{ad}@SCV)}$ values are listed in Table S5.

Table S5. Formation energies (ΔE_{Form}) and dissolution potentials ($U_{Diss(Metal,M_{ad}@SCV)}$) of M_{ad}@SCV catalysts.

	ΔE_{Form} (eV)	$U_{Diss(Metal,M_{ad}@SCV)}$ (V)
Co_{ad}@SCV	-3.42	0.98
Ni_{ad}@SCV	-3.67	1.12
Cu_{ad}@SCV	-0.84	0.20

References

[1] Righi, G., Fabris, S., & Piccinin, S. (2021). Oxygen Evolution Reaction on the Fe_3O_4(001) Surface: Theoretical Insights into the Role of Terminal and Bridging Oxygen Atoms. *The Journal of Physical Chemistry C*, *125*(34), 18752-18761.

[2] Müllner, M., Riva, M., Kraushofer, F., Schmid, M., Parkinson, G. S., Mertens, S. F., & Diebold, U. (2018). Stability and catalytic performance of reconstructed Fe_3O_4(001) and Fe_3O_4 (110) surfaces during oxygen evolution reaction. *The Journal of Physical Chemistry C*, *123*(13), 8304-8311.

[3] Grumelli, D., Wiegmann, T., Barja, S., Reikowski, F., Maroun, F., Allongue, P., Balajka, J., Parkinson, G. S., Diebold, U., Kern, K., & Magnussen, O. M. (2020). Electrochemical stability of the reconstructed Fe_3O_4(001) surface. *Angewandte Chemie International Edition*, *59*(49), 21904-21908.

[4] Back, S., Kulkarni, A. R., & Siahrostami, S. (2018). Single metal atoms anchored in two-dimensional materials: bifunctional catalysts for fuel cell applications. *ChemCatChem*, *10*(14), 3034-3039.
